# The Additive Inflammatory *In Vivo* and *In Vitro* Effects of IL-7 and TSLP in Arthritis Underscore the Therapeutic Rationale for Dual Blockade

**DOI:** 10.1371/journal.pone.0130830

**Published:** 2015-06-25

**Authors:** Maarten R. Hillen, Sarita A. Y. Hartgring, Cynthia R. Willis, Timothy R. D. J. Radstake, Cornelis E. Hack, Floris P. J. G. Lafeber, Joel A. G van Roon

**Affiliations:** 1 Department of Rheumatology & Clinical Immunology, University Medical Center Utrecht, Utrecht, The Netherlands; 2 Laboratory of Translational Immunology, University Medical Center Utrecht, Utrecht, The Netherlands; 3 Inflammation Lab, Amgen Inc., Seattle, Washington, United States of America; Wayne State University, UNITED STATES

## Abstract

**Introduction:**

The cytokines interleukin (IL)-7 and thymic stromal lymphopoietin (TSLP) signal through the IL-7R subunit and play proinflammatory roles in experimental arthritis and rheumatoid arthritis (RA). We evaluated the effect of inhibition of IL-7R- and TSLPR-signalling as well as simultaneous inhibition of IL-7R- and TSLPR-signalling in murine experimental arthritis. In addition, the effects of IL-7 and TSLP in human RA dendritic cell (DC)/T-cell co-cultures were studied.

**Methods:**

Arthritis was induced with proteoglycan in wildtype mice (WT) and in mice deficient for the TSLP receptor subunit (TSLPR-/-). Both mice genotypes were treated with anti-IL-7R or phosphate buffered saline. Arthritis severity was assessed and local and circulating cytokines were measured. Autologous CD1c-positive DCs and CD4 T-cells were isolated from peripheral blood of RA patients and were co-cultured in the presence of IL-7, TSLP or both and proliferation and cytokine production were assessed.

**Results:**

Arthritis severity and immunopathology were decreased in WT mice treated with anti-IL-7R, in TSLPR-/- mice, and the most robustly in TSLPR-/- mice treated with anti-IL-7R. This was associated with strongly decreased levels of IL-17, IL-6 and CD40L. In human DC/T-cell co-cultures, TSLP and IL-7 additively increased T-cell proliferation and production of Th17-associated cytokines, chemokines and tissue destruction factors.

**Conclusion:**

TSLP and IL-7 have an additive effect on the production of Th17-cytokines in a human *in vitro* model, and enhance arthritis in mice linked with enhanced inflammation and immunopathology. As both cytokines signal via the IL-7R, these data urge for IL-7R-targeting to prevent the activity of both cytokines in RA.

## Introduction

Interleukin (IL)-7 is a potent immunostimulatory cytokine of the IL-2 cytokine family that is mainly produced by stromal cells of primary lymphoid organs and plays a pivotal role in T-cell development and homeostasis in mice and humans [1,2]. IL-7 signals via the high affinity IL-7 receptor-alpha chain (IL-7R) in complex with the common gamma chain and mainly acts on T-cells, inducing potent T-cell proliferation and cytokine production. IL-7 enhances inflammation in various autoimmune disorders, including rheumatoid arthritis (RA) where it is increased in the synovial fluid (SF) and stimulates T-cells to produce various cytokines including tumor necrosis factor (TNF)-α, interferon (IFN)-γ, and IL-17 [3,4].

Thymic stromal lymphopoietin (TSLP) is an IL-7-related cytokine that shares the IL-7R for signalling, but only when in complex with the unique TSLP receptor (TSLPR) instead of the common gamma chain [5,6]. It is produced by fibroblasts, epithelial cells, mast cells, smooth muscle cells, macrophages, granulocytes, monocyte-derived DCs, and keratinocytes [7–12]. TSLP acts on classical DCs (cDCs, also referred to as myeloid DCs/mDCs), mast cells, monocytes, granulocytes, natural killer T (NKT)-cells, and activated T-cells [9,13–16]. Like is true for IL-7, TSLP levels are increased in RA SF [17,18]. Whereas IL-7 acts directly on T-cells, TSLP indirectly activates T-cells via stimulation of cDCs, which in turn become extremely potent T-cell activators promoting T-cell proliferation and production of proinflammatory cytokines TNF-α, IFN-γ, and IL-17 in the presence of Th2 mediators [18,19].

Our group previously demonstrated that IL-7 and TSLP separately play a pro-inflammatory role in experimental murine arthritis. Injections with IL-7 in a collagen-induced arthritis (CIA) model induced increased arthritis severity [20] while administering a monoclonal anti-IL-7R antibody in the same model reduced disease severity [21]. TSLPR-deficiency resulted in less severe disease in both CIA and proteoglycan-induced arthritis (PGIA) models along with reduced levels of proinflammatory cytokines, chemokines, and factors involved in tissue destruction [22]. Furthermore, we have shown with *in vitro* assays using cells from RA patients that both cytokines induce production of key proinflammatory factors involved in RA pathogenesis [3,18]. Though both IL-7 and TSLP induce T-cell activation and proinflammatory cytokine production via different target cells while signalling through the same receptor subunit, their combined effects have not been studied in arthritis. We here investigated the effects of simultaneous blocking of TSLP and IL-7 activity in murine experimental arthritis and studied their combined effects in *in vitro* DC + T-cell co-cultures using RA patient material.

## Materials and Methods

### Proteoglycan-induced arthritis

TSLPR deficient (TSLPR-/-) mice were generated as previously described [23] and backcrossed to a BALB/c background. Proteoglycan (PG) was used to induce arthritis (PGIA) in 24-week-old female BALB/c mice (Charles River Laboratories Inc). Human PG was dissolved at 20 mg/mL in phosphate buffered saline (PBS) and emulsified in an equal volume of synthetic adjuvant dimethyl-dioctadecyl-ammoniumbromide (DDA; Sigma) in PBS. On day 0 and day 21, all mice were immunised with 200 μL PG emulsion (400 μg PG and 2 mg DDA) ip. Wildtype and TSLPR-/- mice were divided into two groups of 16 or 17 mice. The groups were treated with either 100 μg of monoclonal rat anti-mouse IL-7R antibody (IgG2b, Amgen Inc. Seattle, WA, USA) or PBS at days 21, 24, 27, 30, and 33. Mice were sacrificed by CO_2_ inhalation at day 36. All animal experiments were approved by the institutional animal care and use committee of Amgen.

### Arthritis assessment

Arthritis symptoms were graded as previously described [20]. In short, arthritis symptoms were graded from day 24 onwards using a scale from 0–4 based on the severity of arthritis. Each limb was graded, giving a maximum possible score of 16. Arthritis incidence was defined as grade 1 or higher. Researchers examining mice for onset and severity of arthritis were blinded to mouse genotype and treatment group.

Radiological joint damage was assessed as previously described [21]. Both ankles of all mice ankles were scored for severity of radiographic lesions by researchers blinded for mouse characteristics, using a scoring system ranging from grade 0 to grade 3. The mean value per ankle was averaged for each mouse.

Histological joint damage was assessed as previously described [20]. Briefly, tissue sections of 5μm were prepared and stained with hematoxilin-eosin (HE). Slides were scored for severity of cellular infiltrates, subchondral bone erosion, osteophyte formation and articular cartilage erosion. For osteoclast evaluation, slides were stained with tartrate-resistant acid phosphatase (TRAP). As control, sections were treated identically without presence of the enzyme substrate, no staining was observed in these sections. Each histological parameter was graded on a scale from 0–4 and the mean value per ankle was averaged for each mouse.

### Splenic and thymic cell preparation and flow cytometry

To check the T-cell compartment in primary lymphoid organs due to TSLPR deficiency and anti-IL-7R administration, spleen and thymus were collected from all mice at day 36. They were weighed and single cell suspension was prepared in Hank’s balanced salt solution (HBSS; Invitrogen, Carlsbad, CA, USA) with 5% fetal bovine serum (FBS; Invitrogen) using a 70 μm cell strainer (Falcon, BD Bioscience, San Jose, CA, USA). Red blood cells in spleen were lyzed by 5 minute incubation in lysis buffer (Invitrogen). The reaction was stopped by addition of 10 mL cold PBS. Cells were counted, pelleted and resuspended in HBSS with 5% FBS and 1 μg Fc block (2.4G2; CD32/16, BD). Cells were incubated with monoclonal anti-CD4 APC/Cy7 (clone Gk1.5; BD), anti-CD8 Pacific Blue (clone 53–6.7; BD), anti-CD19 PerCP/Cy 5.5 (clone 1D3; BD), anti-CD44 PerCP/Cy 5.5 (clone IM7; eBioscience), and anti-CD62L APC (clone mel-14; BD). 10^5^ events were registered using FACS-LSRII (BD). Events were gated for viable lymphocytes based on forward and side scatter and specific staining using FlowJo software (Tree Star Inc, Ashland, OR, USA).

### Paw lysate and serum multi analyte profiling

Blood samples were taken on day 36 by heart puncture. Additionally, both front paws of each mouse were collected and frozen in liquid nitrogen directly upon removal. Skin was removed and paws were homogenized in a 50 mM Tris-HCL buffer supplemented with 0.1 M NaCL and Triton X-100 (pH 7.4) and mini-EDTA free protease-inhibitor tablets (Roche) using a tissue lyser (Qiagen, Valencia, CA, USA) and 5 mm stainless steel beads (Qiagen). Lysates were stored at -20°C until further analysis. Protein content of the lysates was measured with Pierce BCA total protein quantitation kit and each sample was brought to a total protein content of 1 mg/mL. For 10 mice per group with representative arthritis scores, luminex-based multi analyte profiling (MAP) was performed in the paw protein lysates and in the serum samples by Rules Base Medicine Inc. (Austin, TX, USA) using RodentMAP v2.0. IL-17 was measured with luminex.

### RA patients

RA patients were classified according to the American College of Rheumatology criteria [24]. The University Medical Center Utrecht approved the *in vitro* experiments with human material in compliance with the Helsinki Declaration. All patients gave their written informed consent.

### Cell isolation

Mononuclear cells (MCs) from RA patients were isolated from heparinized peripheral blood (PB) by density centrifugation using Ficoll-Paque Plus (GE Healthcare, Uppsala, Sweden). Prior to MC isolation, PB was diluted 1:1 in RPMI 1640 medium (Gibco, Life Technologies, New York, USA) containing penicillin (100 U/mL), streptomycin (100 μg/mL) and glutamine (2 mM) (all PAA Laboratories, Pasching, Australia). CD19^−^ CD1c^+^ cDCs and CD4^+^ T-cells were isolated from PB MCs by magnetic-activated cell sorting (MACS) using CD1c (BDCA-1)^+^ and CD4^+^ isolation kits (Miltenyi Biotec, Bergisch Gladbach, Germany) according to manufacturer’s instructions.

### DC/T-cell co-cultures

Cells were cultured in RPMI glutamax (Gibco) supplemented with penicillin, streptomycin, and 10%, v/v, human AB serum (GemCell, West Sacramento, USA). Isolated CD1c-positive cDCs (2000–5000 cells/well) were co-cultured with 50.000 autologous CD4 T-cells per well in round-bottomed 96-well plates in the presence of 20ng/ml recombinant TSLP (R&D systems) and 0.3–10 ng/mL of recombinant IL-7 (Preprotech Inc, Rocky Hill, NJ, USA) where appropriate. Cells were cultured for 6 days at 37°C and proliferation and cytokine production were measured. Proliferation was measured (n = 7) by ^3^H-Thymidine incorporation assay at the end of the culture period. ^3^H-Thymidine (1μCi/well; PerkinElmer, Waltham, USA) was added during the last 18 hours of the culture period. In separate cultures (n = 7), supernatants of co-cultured T-cells were re-stimulated with ionomycin (500ng/ml) and phorbol myristate acetate (50ng/ml) (both from Sigma-Aldrich) during the last 24 hours of the culture period. Culture supernatants were collected and frozen at -80°C until cytokine production analysis with Luminex.

### Cytokine analysis

Cytokine production of human *in vitro* cultures by multiplex immunoassays were performed at the MultiPlex Core Facility of the Laboratory for Translational Immunology (UMC Utrecht, Netherlands) using an in-house validated panel of analytes. Uniquely color-coded magnetic beads (MagPlex Microsphere, Luminex, Austin, Texas, USA) were conjugated to antibodies specific for the reported analytes and incubated with 50 uL of standard dilutions or samples for 1 hour (with continuous shaking in he dark). Plates were washed (Bio-Plex Pro II Wash Station; Bio-Rad, Hercules, California, USA) and a corresponding cocktail of biotinylated detection antibodies was added for 1 hour. Repeated washings were followed by a 10 minute streptavidin-phycoerythin (PE) incubation. Fluorescence intensity of PE was measured using a Flexmap 3D system (Luminex) and analyzed using Bio-Plex Manager software version 6.1 (Biorad) using 5-parameter curve fitting.

### Statistical Analyses

For mouse experiments, Mann-Whitney U test with two-sided testing was used to examine differences between treatment and control groups of mice in arthritis score, radiographic- and histological joint scores. Pearson Chi-Square test was used for comparing arthritis incidence between treatment groups. For MAP data analysis, Kruskal Wallis test with post-hoc Mann Whitney U test using two-sided testing was used. For *in vitro* human proliferation experiments, Mann Whitney U test with two-sides testing was used. For human cytokine expression experiments, Mann Whitney U test with one-sided testing was used. P-values of 0.05 or smaller were considered statistically significant.

## Results

### Blockade IL-7- and TSLP signalling significantly decreases severity of arthritis

**Fig 1:**
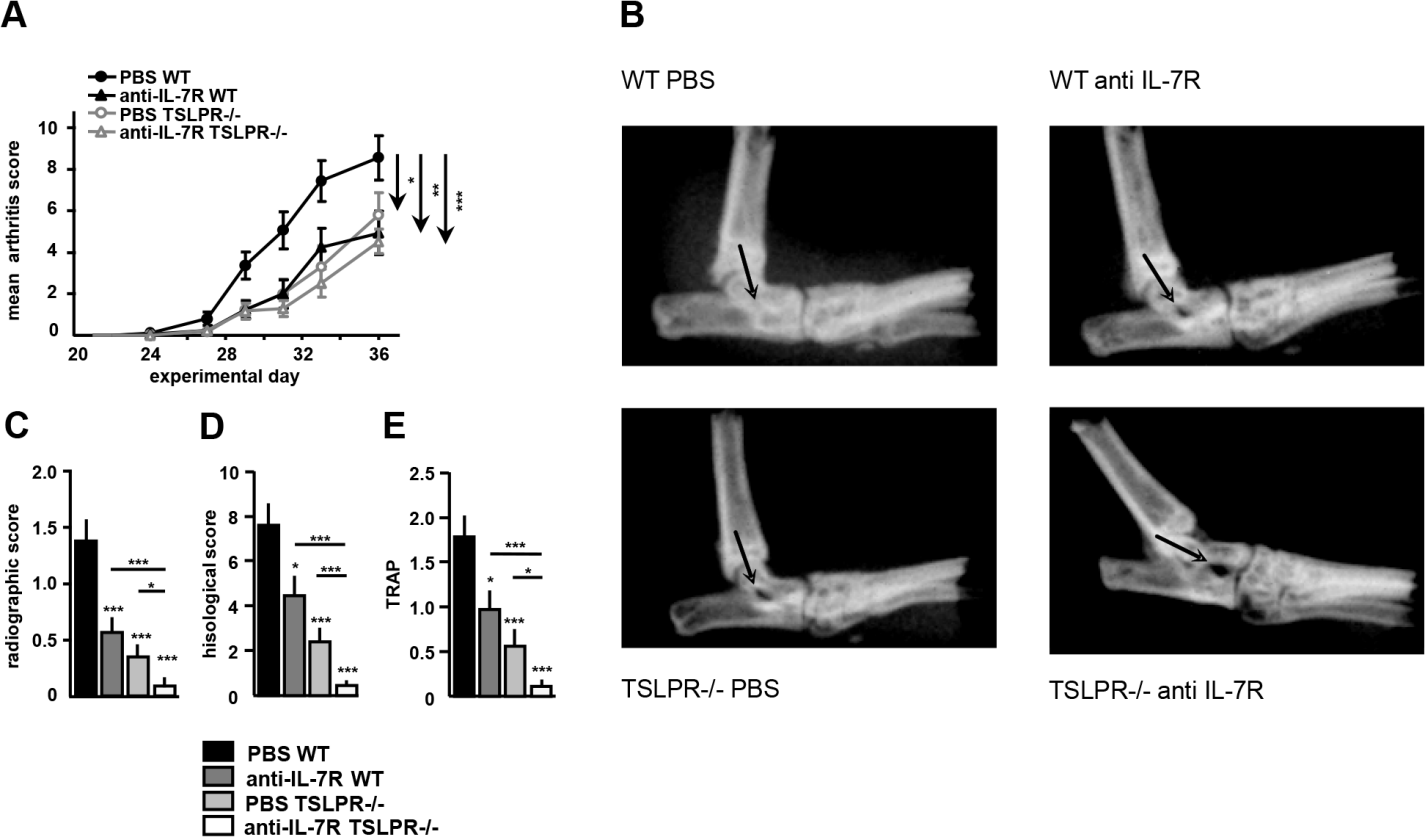
Arthritis severity is inhibited in TSLPR deficient mice and upon blockade of the IL-7R. WT or TSLPR-/- mice were treated with PBS or anti IL-7Rα antibodies (anti IL-7R, 100 μg ip. on day 21, 24, 27, 30, and 33) and arthritis severity was graded by visual examination of the paws. IL-7R blockade, TSLPR deficiency or a combination of both significantly decreased arthritis severity compared to PBS WT mice (A). The same was true for radiological joint damage (B, C), histology (D) and osteoclast formation as measured by the number of TRAP+ cells (E). Anti-IL-7R TSLPR-/- mice showed almost complete inhibition of joint damage. Values are mean ± SEM of 16 mice per group. *, **, and *** indicate statistical differences of p<0.05, p<0.01, or p<0.005 respectively.

Treatment of WT mice with anti-IL-7R (25 ± 5.9, p = 0.008; Mean area under curve ± SEM) or TSLPR-/- mice treated with PBS (24.1 ± 6.0, p = 0.019) significantly reduced clinical arthritis score compared to PBS treated WT mice (50.7 ± 6.8). Strikingly, TSLPR-/- mice treated with anti-IL-7R displayed even lower clinical arthritis scores compared to the anti-IL-7R treated WT mice and the PBS treated TSLPR-/- mice (18.7 ± 3.6, p = 0.003), though there was no statistically significant difference between anti-IL-7R treated TSLPR-/- mice and the anti-IL-7R treated WT mice or PBS treated TSLPR-/- mice (Fig 1A).

Anti-IL-7R treated WT mice (0.6 ± 0.1, p = 0.002) and PBS treated TSLPR-/- mice (0.3 ± 0.1, p<0.001) showed a significantly decreased radiographic joint damage score per ankle compared to PBS treated WT mice (Mean ± SEM; 1.4 ± 0.2). In-line with the observed clinical arthritis scores, the anti-IL-7R treated TSLPR-/- mice showed an even lower radiological score (0.1 ± 0.1; p<0.001), which was significantly lower than the scores in all three other groups (Fig 1B and 1C). This was confirmed with histopathological analysis (S1 Fig), demonstrating significantly decreased joint damage in anti-IL-7R treated TSLPR-/- mice compared to all three other groups (Fig 1D). The same holds true for osteoclast activity as measured by TRAP staining (Fig 1E).

The arthritis incidence at day 36 was similar in all of the groups. However, there seemed to be a delay in disease onset in all three experimental groups as they showed significantly lower arthritis incidence during observation days 27–31 compared to the PBS treated WT mice control group (S2 Fig).

### IL-7R blockade and TSLPR-deficiency alter numbers of thymic and splenic T-cells in mice

**Fig 2:**
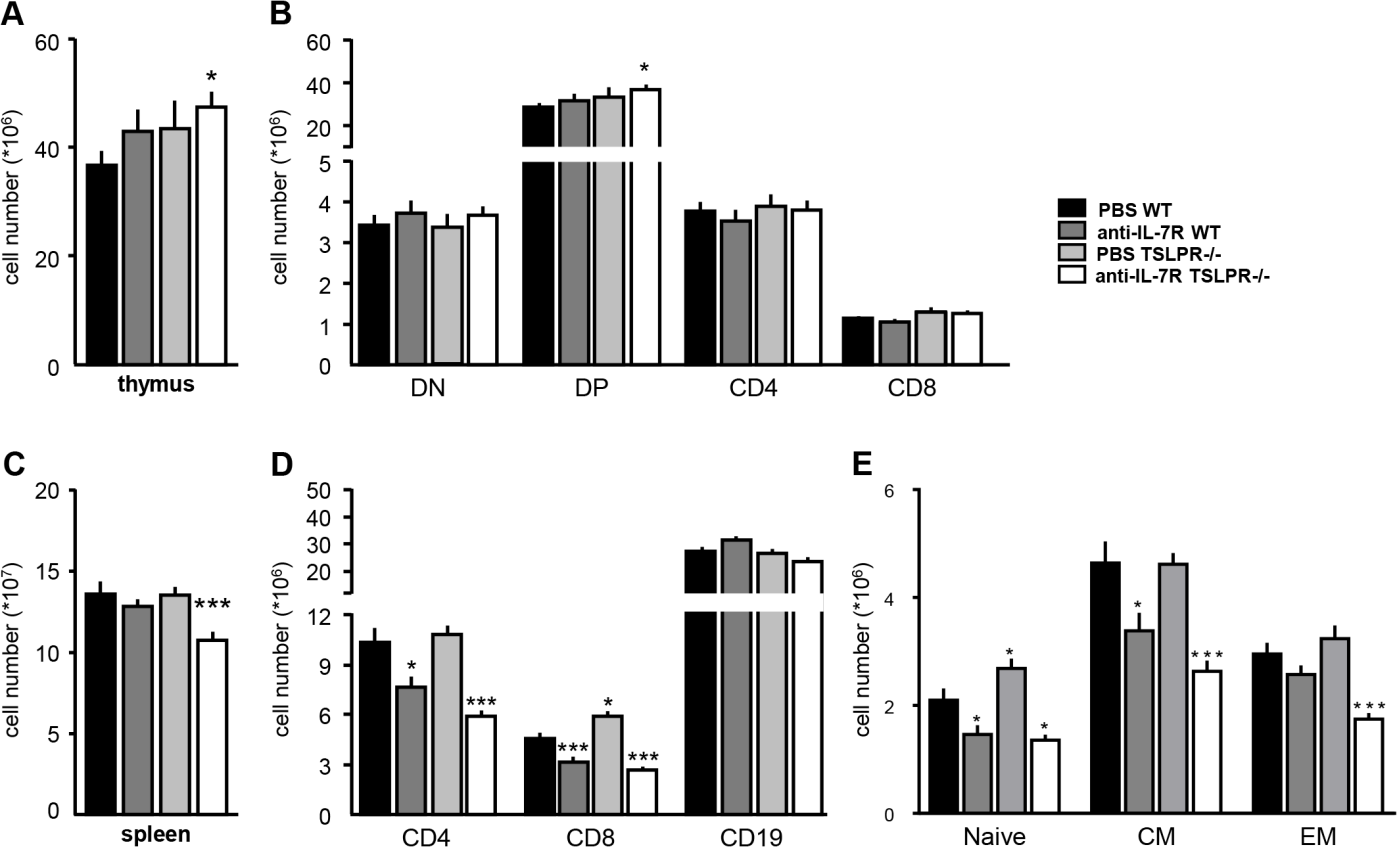
TSLPR-deficiency and IL-7R blockade alter numbers of thymocytes and splenocytes. Total numbers of thymocytes were modestly increased in anti-IL-7R treated TSLPR-/- mice as compared to PBS treated WT mice (A), associated with an increase in CD4+ CD8+ double positive (DP) thymocytes (B). Total numbers of splenocytes were decreased in anti-IL-7R treated TSLPR-/- mice compared to PBS treated WT mice (C), associated with a decrease in CD4+ and CD8+ T-cells in the anti-IL-7R treated TSLPR-/- mice (D). The number of CD4+ T-cells with characteristics of naive (Naive; CD44-CD62L+), central memory (CM; CD44+CD62L+) and effector memory (EM; CD44+CD62L-) T-cells were significantly decreased in anti-IL-7R TSLPR-/- mice as compared to PBS WT mice. Values are mean ± SEM of 16 mice per group. * and *** indicate statistical differences of p<0.05 and p<0.005 respectively.

As IL-7 and TSLP have been shown to affect T- and B-cell expansion [2,19,25], we investigated the effects of anti-IL-7R treatment and TSLPR deficiency on the number of T- and B-cells in spleen and thymus. Anti-IL-7R treatment and TSLPR deficiency separately did not have a significant effect on the number of thymic and splenic lymphocytes. However, anti-IL-7R treated TSLPR-/- mice showed significantly increased total number of thymocytes compared to PBS treated WT mice (Fig 2A), which was associated with an increase in CD4^+^CD8^+^ double-positive thymocytes (Fig 2B). The other thymocyte subsets were not different between any of the groups. In the spleens of anti-IL-7R treated TSLPR-/- mice, a decrease in total cell number was observed compared to PBS treated WT mice (Fig 2C), associated with lower CD4^+^ T-cell and CD8^+^ T-cell numbers (Fig 2D). In the anti-IL-7R treated WT mice, numbers of both CD4 and CD8 T-cells were lower compared to PBS treated WT mice. In the TSLPR-/- mice, only the number of CD8^+^ T-cells was higher compared to WT mice. Splenic B-cell numbers were not different between any of the groups.

In the spleen, naïve (CD44^-^ CD62L^+^) and central memory (CD44^+^ CD62L^+^) CD4 T-cell numbers were decreased in the anti-IL-7R treated WT mice compared to PBS treated WT mice (Fig 2E). In the anti-IL-7R treated TSLPR-/- mice, the frequencies of naïve, central memory and effector memory (CD44^+^ CD62L^-^) CD4 T-cells were decreased compared to PBS treated WT mice. In contrast, PBS treated TSLPR-/- mice showed higher numbers of naïve CD4 T-cells compared to PBS treated WT mice (Fig 2E).

### Prevention of IL-7R and TSLPR signalling reduces expression of local and systemic pro-inflammatory mediators

**Fig 3:**
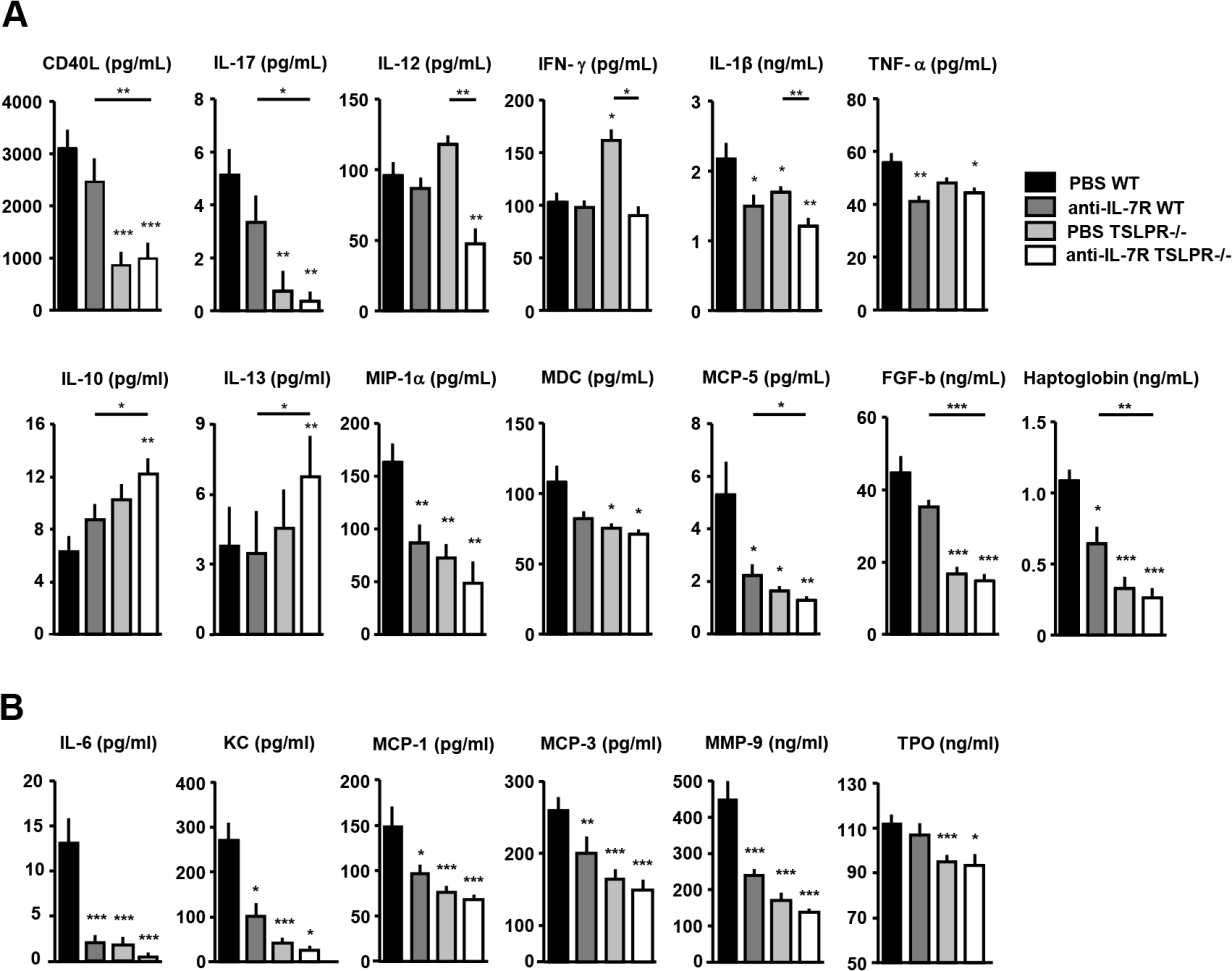
IL-7R blockade and TSLPR-deficiency additively reduce production of pro-inflammatory cytokines, chemokines and mediators involved in tissue destruction and angiogenesis. Cytokine concentrations in paw protein lysates (A) and serum (B) were measured by multicytokine analysis. Cytokines associated with T-cell and monocyte/macrophage activation, chemotaxis, angiogenesis and tissue destruction were reduced by IL-7R blockade and TSLPR deficiency. Values are mean ± SEM of 10 mice per group with representative arthritis scores. *, **, and *** indicate statistical differences of p<0.05, p<0.01, or p<0.005 respectively.

Multi-cytokine analysis was performed on paw lysates and serum from all four groups studied. Compared to the PBS treated WT control group, the anti-IL-7R treated TSLPR-/- mice showed decreased CD40L, IL-17 and IL-12 levels in the paw lysates (Fig 3A) coinciding with lower IL-6 serum levels (Fig 3B). Regulatory cytokines IL-10 and IL-13 were significantly increased in the anti-IL-7R treated TSLPR-/- mice. In addition, significant decreases in IL-1β and chemokines MIP-1α, MDC, MCP-1, MCP-3, MCP-5 and KC were observed upon combined prevention of IL-7R and TSLPR signalling, whereas IFN-γ did not show a difference in paw lysates or serum. Furthermore, significant decreases of factors involved in tissue destruction FGF-b, TPO and MMP-9 were found (Fig 3A and 3B). IL-4 in all cases was not measurable (data not shown).

### IL-7 and TSLP additively induce T-cell proliferation and Th17-associated cytokine production in cDC/T-cell co-culture of human primary cells

**Fig 4:**
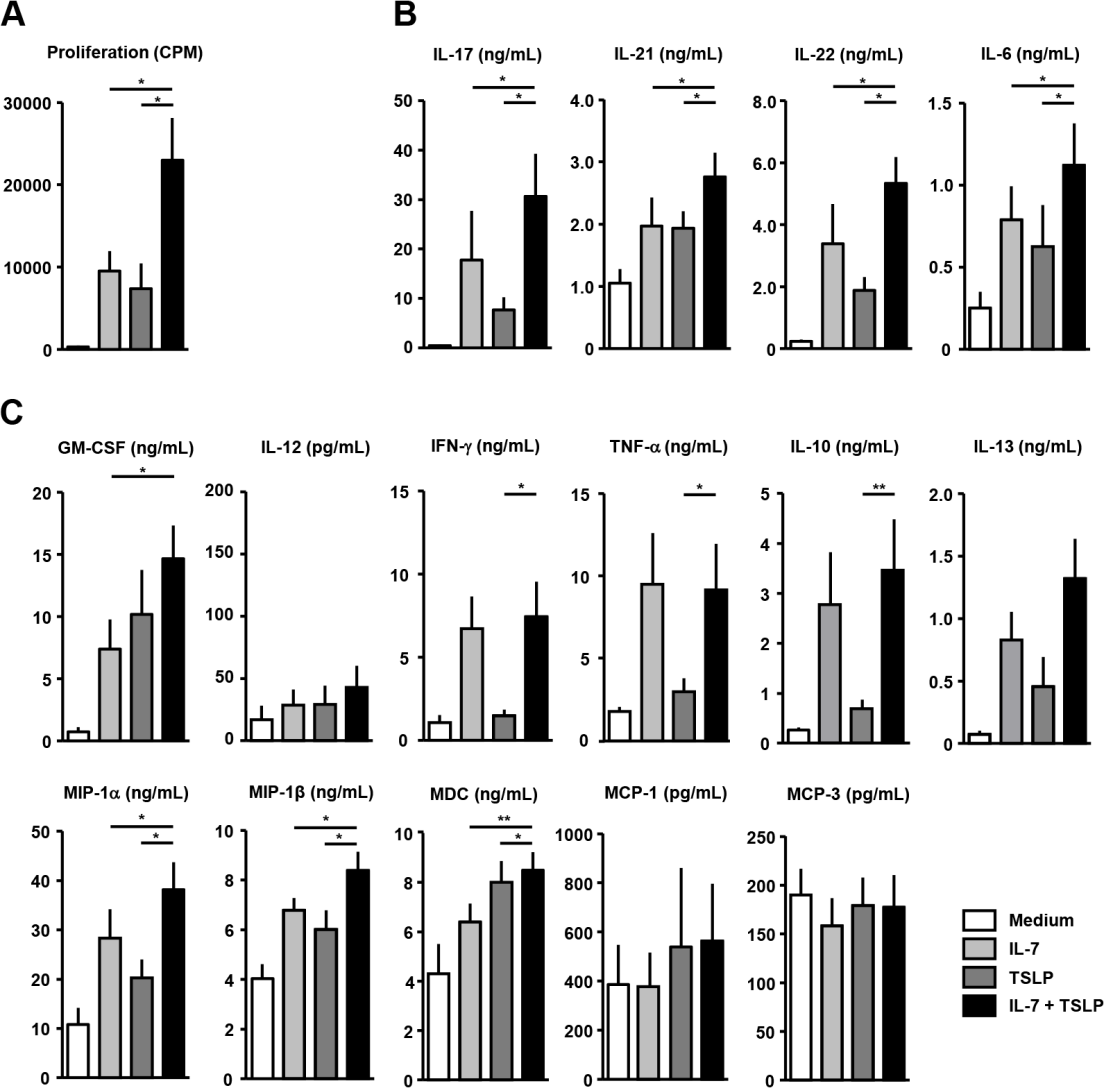
TSLP and IL-7 additively induce T-cell proliferation and Th17-activity in a human cDC/T-cell co-culture. Paired CD1c-expressing classical dendritic cells (cDCs) and CD4^+^ T-cells were isolated from rheumatoid arthritis patients. CD4 T-cells were co-cultured with cDCs for 6 days in the presence of thymic stromal lymphopoietin (TSLP), IL-7 or both cytokines. TSLP and IL-7 additively induce T-cell proliferation as measured with tritium thymidine incorporation (A). TSLP and IL-7 show additive induction of Th17-associated cytokines (B). Additive effects were also found on expression of other proinflammatory cytokines and T-cell attracting chemokines (C). * and ** indicate statistical differences of p<0.05 and p<0.01 respectively.

To study whether the combined effects of IL-7 and TSLP observed in our mouse model are also relevant in the human setting, we performed co-cultures with autologous CD1c^+^ cDCs and CD4^+^ T-cells, the main target cells for TSLP and IL-7 respectively. Combined addition of both IL-7 and TSLP significantly increased T-cell proliferation compared to either cytokine alone (Fig 4A). IL-7 and TSLP significantly and additively increased secretion of Th17-associated cytokines IL-17, IL-21, IL-22 and IL-6 compared to either cytokine alone (Fig 4B). In addition, T-cell attracting chemokines MIP-1α, MIP-1β and MDC were additively upregulated by IL-7 and TSLP compared to either cytokine alone (Fig 4C). Furthermore, a range of other mediators showed a stronger increase in the presence of both cytokines compared to either cytokine alone, (Fig 4C). Regulatory cytokines IL-10 and IL-13 (Fig 4C) and IL-4 and IL-5 (not shown) were not significantly additively modulated.

## Discussion

Here we show that combined ablation of IL-7R and TSLPR signalling strongly inhibits experimental arthritis and almost completely prevents immunopathology. This is associated with marked decreases in T-cell attracting- and co-stimulatory factors and levels of cytokines associated with Th17 activity. We further observed robust additive effects of IL-7 and TSLP on T-cell proliferation and Th17 activation in human DC/T-cell *in vitro* co-cultures, suggesting these data are relevant for the human setting.

In the anti-IL-7R treated TSLPR-/- mice, strongly ameliorated clinical arthritis and immunopathology were associated with decreased levels of IL-17-associated cytokines IL-17 and IL-1β locally and IL-6 systemically. This is consistent with previous data that describe PGIA to be strongly IL-17 dependent, especially in the later stages of disease [26]. As CD40L can trigger production of IL-17 by T-cells [27], the observed local decrease in CD40L may contribute to disease amelioration. In line with these mouse data, in the DC/T-cell co-cultures we observed potent additive effects of IL-7 and TSLP on production of Th17-associated cytokines IL-17, IL-21 and IL-22 that can strongly stimulate autoimmunity [28]. Though we have added the cytokines in our *in vitro* cultures while the *in vivo* model is based on abrogation of signalling, the effects on Th17-activity and immune activation are clear in both settings. This indicates that the additive effects of TSLP and IL-7 are not restricted to the mouse setting and underscores the potential of their combined blockade in patients.

The anti-IL-7R antibody used in our mouse experiments could potentially inhibit TSLP signalling in addition to IL-7 signalling, but we have shown *in vitro* that the antibody is 100-fold more effective at blocking IL-7-induced chemokine secretion by murine DCs compared to TSLP-induced secretion (C.R. Willis, unpublished data). Additionally, the differential effect of anti-IL-7R antibody treatment and TSLPR-deficiency on thymic- and splenic cell numbers as well as expression of IFN-γ and IL-12 production strongly suggests a selective inhibition of IL-7-mediated immune responses. Though we have previously observed a larger decrease in thymocytes and splenocytes upon treatment with anti-IL-7R [21], the previous study was performed in young mice while the present study was performed in retired breeders of at least 6 months old. At this age the number of IL-7-producing stromal cells in thymus and bone marrow is dramatically reduced compared to young mice [29,30], which likely causes the less pronounced effects of blockade of IL-7 signalling on T-cell numbers that we observed in this study.

In mice TSLPR-deficiency results in strong reduction of IL-4 production [31]. We could not verify this as IL-4 was not detectable in our study. We did observe an increase in Th1 mediators IFN-γ and IL-12 in TSLPR-deficient mice. Though Th1-activity is known to promote PGIA, IFN-γ and IL-12 have also been demonstrated to down regulate Th17 activity [32,33], thus their increased levels may contribute to the marked decrease in IL-17 production we observe in the TSLPR-deficient mice. In addition, the enhanced IFN-γ and IL-12 production in deficient mice was negated by anti-IL-7R treatment, which also further down regulated IL-17 expression.

TNF-α levels were modestly but significantly inhibited upon anti-IL-7R treatment in the mice. This is in line with our DC/T-cell co-cultures, where IL-7 upregulated TNF-α secretion. TSLP deficiency only minimally decreased TNF-α levels in our mice and TSLP induced expression of TNF-α to limited extent in the DC/T-cell co-cultures. However, in short-term (24 hours) DC-priming experiments we previously showed that TSLP-stimulated cDCs clearly produce TNF-α and are able to induce TNF-α production by T-cells [34]. The cause of the less evident inhibition of TNF-α in mice in the present study is unclear, but may be related to species differences. Redundancy in arthritogenic cytokines could also play an important role, with IL-1β taking over the role of TNF-α as local IL-1β concentrations were much higher than TNF-α.

A significant increase in regulatory cytokines IL-10 and IL-13 was only observed in the anti-IL-7R treated TSLPR-/- mice as compared to PBS treated WT control mice. Considering the inhibitory potential of these cytokines on many proinflammatory mediators like IL-17, IL-6 and IL-1β, they may contribute to the observed suppression of arthritis severity [35]. As IL-10 and IL-13 were also produced in the DC/T-cell co-cultures, these cells might be a major source of these cytokines in the mice as well, contributing to disease amelioration.

This is the first study to assess the combined roles of IL-7 and TSLP in autoimmunity and shows that both cytokines play a specific pro-inflammatory role during experimental arthritis. This emphasizes the additional benefits of TSLP-signalling blockade in conjunction with blockade of IL-7-signalling as a therapeutic strategy in rheumatoid arthritis and possibly other autoimmune diseases. As both cytokines signal via the IL-7R, the data described here emphasize the potential for antibodies or other compounds that target this subunit or abrogate activity of both cytokines simultaneously to prevent immunopathology and tissue damage in RA. Recently developed second-generation anti-IL-7R antibodies that inhibit signalling of both IL-7 and TSLP may prove to be a very beneficial treatment option.

## Supporting Information

S1 Fig Joint damage and infiltration are inhibited in TSLPR deficient mice and upon blockade of the IL-7R. Photomicrographs of representative ankle joint sections. TSLPR deficiency and IL-7R blockade decrease the destruction of the articular surface and the amount of infiltrating cells in the connective tissue and joint space (arrows). Original magnification x40.
(TIF)

S2 Fig Abrogation of IL-7 and TSLP signalling results in delayed arthritis development. WT or TSLPR-/- Balb/c mice were treated with PBS or anti-IL-7Rα antibodies on day 21, 24, 27, 30, and 33. Arthritis severity was graded and arthritis incidence was defined as grade 1 or higher. On day 31 all three groups showed lower arthritis incidence as compared to the PBS treated WT mice. * indicates a statistical difference of p<0.05.
(TIF)
