# Supplementary figures and images for: The Additive Inflammatory In Vivo and In Vitro Effects of IL-7 and TSLP in Arthritis Underscore the Therapeutic Rationale for Dual Blockade
Source: PLoS One. 2015 Jun 25;10(6):e0130830. doi: 10.1371/journal.pone.0130830 (PMC4482403; doi:10.1371/journal.pone.0130830)

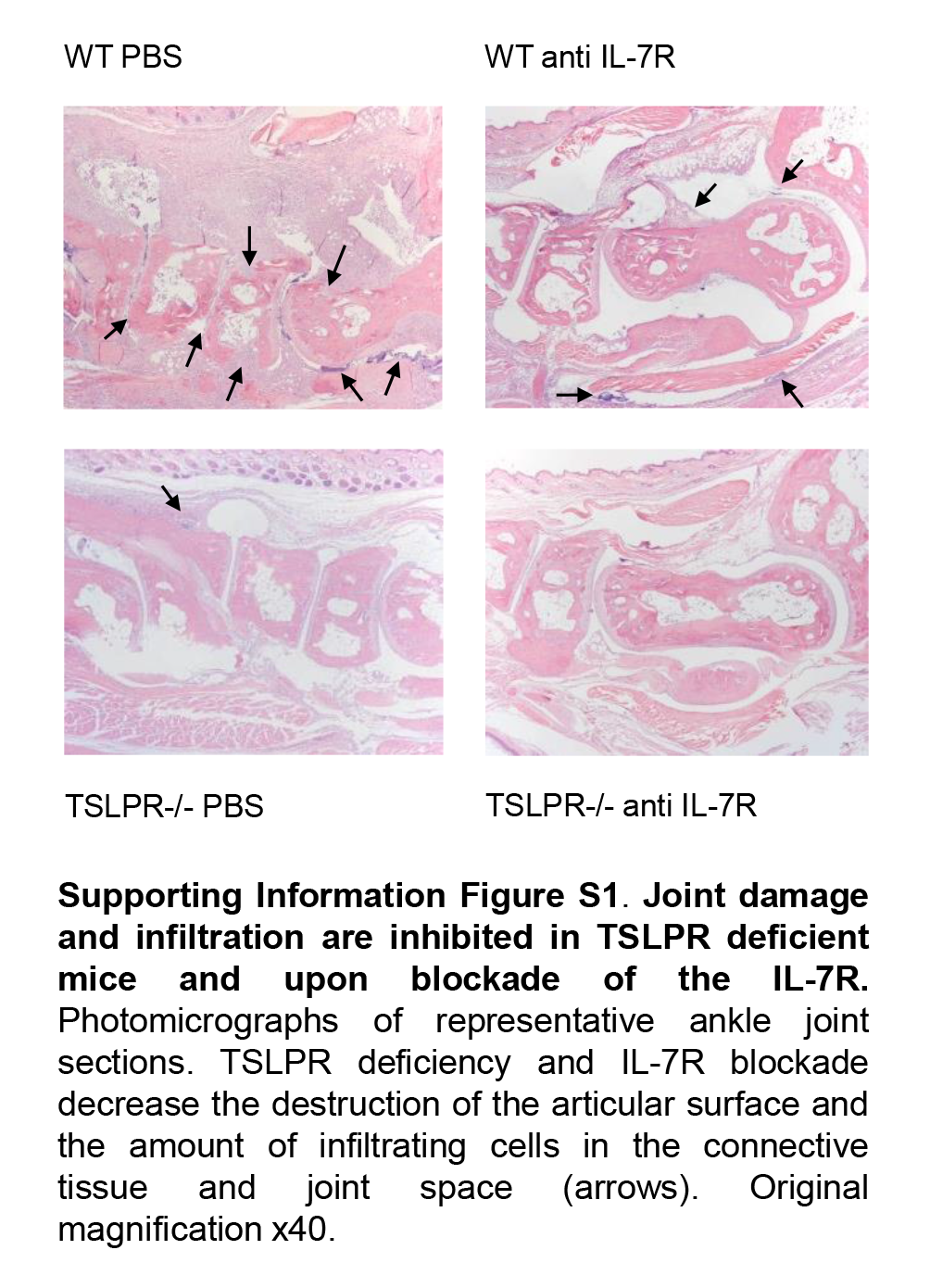

Supplement: S1 Fig — Photomicrographs of representative ankle joint sections. TSLPR deficiency and IL-7R blockade decrease the destruction of the articular surface and the amount of infiltrating cells in the connective tissue and joint space (arrows). Original magnification x40. (TIF) [file pone.0130830.s001.tif]

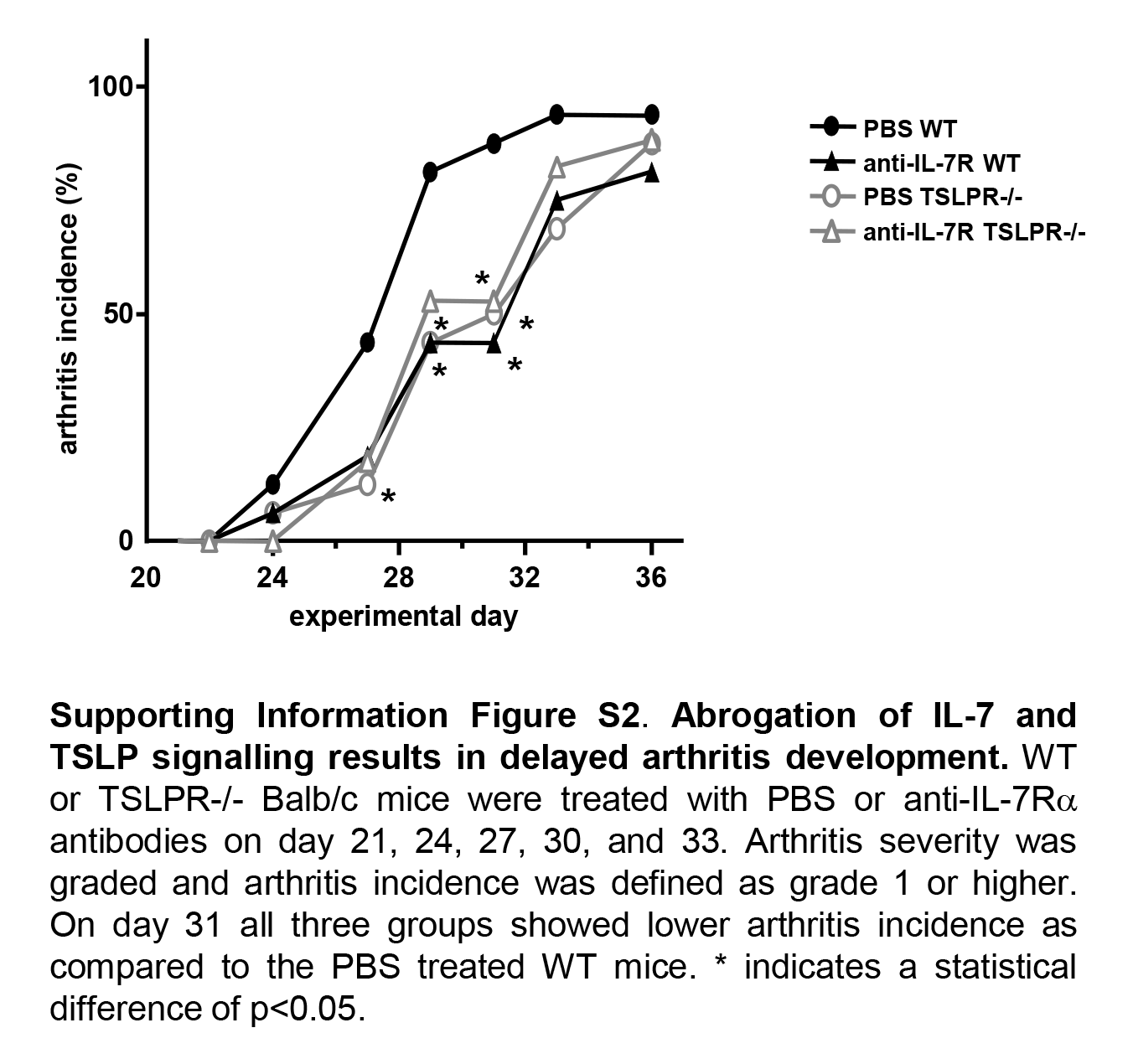

Supplement: S2 Fig — WT or TSLPR-/- Balb/c mice were treated with PBS or anti-IL-7Rα antibodies on day 21, 24, 27, 30, and 33. Arthritis severity was graded and arthritis incidence was defined as grade 1 or higher. On day 31 all three groups showed lower arthritis incidence as compared to the PBS treated WT mice. * indicates a statistical difference of p<0.05. (TIF) [file pone.0130830.s002.tif]
